# Inducing Behavioral Change in Seekers of Pro-Anorexia Content Using Internet Advertisements: Randomized Controlled Trial

**DOI:** 10.2196/mental.8212

**Published:** 2018-02-22

**Authors:** Elad Yom-Tov, Anat Brunstein-Klomek, Or Mandel, Arie Hadas, Silvana Fennig

**Affiliations:** ^1^ Microsoft Research Herzliya Israel; ^2^ Department of Child and Adolescent Psychiatry Schneider Children's Medical Center of Israel Petach Tikvah Israel; ^3^ School of Psychology Interdisciplinary Center Herzliya Israel; ^4^ Sackler School of Medicine Tel Aviv University Tel Aviv Israel

**Keywords:** anorexia nervosa, bulimia nervosa, eating disorders, online advertising, online behavior, Pro-ana

## Abstract

**Background:**

The influence of pro-anorexia (pro-ana) websites is debated, with studies indicating both negative and positive effects, as well as significant variation in the effects of different websites for those suffering from eating disorders (EDs) and the general population. Online advertising, known to induce behavioral change both online and in the physical world, has not been used so far to modify the search behavior of people seeking pro-ana content.

**Objective:**

The objective of this randomized controlled trial (RCT) was to examine if online advertisements (ads) can change online search behaviors of users who are looking for online pro-ana content.

**Methods:**

Using the Bing Ads system, we conducted an RCT to randomly expose the searchers for pro-ana content to 10 different ads referring people to one of the three websites: the National Eating Disorders Association, the National Institutes of Mental Health, and MyProAna. MyProAna is a pro-ana website that was found in a previous study to be associated with less pathological online behaviors than other pro-ana websites. We followed participants exposed and unexposed to the ads to explore their past and future online searches. The ads were shown 25,554 times and clicked on 217 times.

**Results:**

Exposure to the ads was associated with a decrease in searches for pro-ana and self-harm content. Reductions were greatest among those referred to MyProAna (reduction of 34.0% [73/215] and 37.2% [80/215] for pro-ana and self-harm, respectively) compared with users who were referred elsewhere (reduction of 15.47% [410/2650] and 3.21% [85/2650], respectively), and with users who were not shown the ads, who increased their behaviors (increase of 57.12% [6462/11,314] and 4.07% [461/11,314], respectively). In addition, those referred to MyProAna increased their search for treatment, as did control users, who did so to a lesser extent. However, users referred elsewhere decreased their searches for this content.

**Conclusions:**

We found that referring users interested in ED-related content to specific pro-ana communities might lessen their maladaptive online search behavior. This suggests that those who are preoccupied with EDs can be redirected to less pathological online searches through appropriate pathways.

**Trial Registration:**

ClinicalTrials.gov NCT03439553; https://clinicaltrials.gov/show/NCT03439553 (Archived by WebCite at http://www.webcitation.org/6xNYnxYlw)

## Introduction

Nowadays, individuals interested in extreme weight loss can find an extensive body of knowledge, as well as support, in their disordered eating through websites colloquially known as *pro-ana* sites. Some of these sites serve as online communities that promote an anorexic lifestyle, mainly by propagating positive viewpoints of anorexia as well as sharing practical tips on how to lose weight and conceal symptoms [1-3]. Daga et al [4] counted at least 300,000 websites promoting anorexic behaviors (257,000 “pro anorexia,” 18,600 “pro-ana,” 14,200 “thinspiration,” and 577 “anorexic-nation”). Studies examining the effects of pro-ana websites have found negative outcomes on their visitors, such as lower self-esteem, higher perceived body weight, and increased likelihood of engaging in weight-restrictive practices [1,5-8]. Online behaviors of those interested in pro-ana sites may include online language usage (posts) and queries [9], as well as drug seeking, smoking, self-injury, and suicide [8].

On the other hand, some studies have suggested that pro-ana websites can also increase subjective feelings of social support, acceptance, and belongingness [10-12]. Therefore, it seems that these sites are not only harmful but may also offer their participants a potential benefit [13,14]. The exact influence of these sites is therefore still not clear, and there seems to be a difference between users who suffer from eating disorders (EDs) and the general population; students exposed to the pro-ana websites had greater negative affect, lower social self-esteem, and lower appearance self-efficacy [5]. *Recovery sites* are less numerous [4] and provide information about the disorder and how to reach treatment. They usually include posts about personal experiences, links to professional sites, forums, and other pages related to EDs. Like *pro-ana* users, pro-recovery visitors are characterized by a similar need of sharing and understanding; they often feel that the virtual forum is more supportive than traditional therapy [15]. Although it is important to provide information and support, the quality of Web-based treatment information at the recovery sites was found to be poor and deficient in accountability [16].

There are also a number of promising alternative Internet-based delivery systems for the treatment of ED patients, although most are still in an early phase of development [17,18]. A previous study, for example, has examined Internet-delivered, computer-assisted health education programs aimed at improving body satisfaction and decreasing the weight and shape concerns [19].

In a previous study, Yom Tov et al showed that there is variance between different pro-ana communities [9], and MyProAna website was found to be associated with the least harmful behaviors of those tested and the highest percentage of treatment seeking among its users. As this previous result was only based on an association rather than a causal relationship, in this study, we investigated how referring users to MyProAna community, using online advertisements (ads), would affect their online search behavior.

The effectiveness of Web advertising has long been investigated, especially due to their prominent role in the context of sales and marketing [20-22]. An important advantage of online advertising is the ability to perform behavioral targeting (BT), a widespread technology in which the ads are shown only to a specific audience to whom they are relevant [22]. Accumulating evidence indicates that online ads can be used to encourage the abandonment of unhealthy practices and, thus, to promote healthier lifestyles. Recently, Yom-Tov et al [23] used BT to present smokers, identified by their online behavior, with different antismoking ads. They then compared the smokers’ searches before and after the intervention and found that the ads made a behavioral change in a gender-dependent manner. To the best of our knowledge, no previous studies have used such online ads to change users’ search behavior in the context of EDs.

Here, we use search behavior to inform of user’s behavior and its reflection of real-world behaviors. Online behavior may reflect offline behavior, including aspects of health, such as taking of prescription drugs, the conditions experienced by people [24], precursors to the development of disease [25], and symptoms indicative of certain cancers [26]. Thus, the ability to change online search behavior is expected to be advantageous in helping to modify behavior in the physical world.

In this study, we used an online randomized control trial (RCT) design [21] to redirect Web users interested in ED to a variety of ED-related websites, including MyProAna community, found to be associated with less harmful online behaviors [9]. First, we identified users interested in ED-related content. Then, we measured whether those users’ online behavior was affected by the presentation of online ads that route the users either to the MyProAna community or to official health informational sites (which are all pro-recovery sites). These were chosen because they are official sites that promote information about EDs and professional treatments. However, in a study among undergraduate students, the authors did not find significant differences in terms of abnormal eating behaviors among people who visited pro-ana sites and those who visited professional EDs sites [6]. Our hypothesis was that participants redirected to the MyProAna community, which was associated with less pathological online behaviors, would show decreased search behavior for other pro-ana communities or to other sites associated with self-harm compared with participants not exposed to our ads or to prorecovery websites, such as the Centers for Disease Control and Prevention (CDC) website.

## Methods

### Overview

We conducted an RCT using the Bing search engine. Users of this search engine who were searching for pro-ana content were randomized into either being shown one of the 10 randomly chosen textual ads, referring people to one of the three randomly chosen websites, or not to be shown these ads. We then followed people, both those exposed to the ads and those not exposed to them, to explore their future searches online. The control intervention was “usual care,” meaning users were served whatever ads the Bing Ads system would have otherwise served. The authors designed the textual ads. The ethical question in the Internet research field of EDs is complicated and remains a continuing dilemma: do we stay passive observers of a social phenomenon that is impossible to control or can we study the field through the active anonymous participation of Internet users. We consider this study ethical as the criteria for the selection of the target population of our study was restricted to people already searching for the pro-ana websites. As the users were already searching for pro-ana content online, we assume they would have arrived at the MyProAna website or similar sites regardless of our intervention. These online communities were extensively studied in the past and were found to have some supportive effects on the users [8-10]. From all the pro-ana websites, we chose the one that was, as per our previous study, associated with the least harmful behaviors [9]. The Microsoft Institutional Review Board and the Interdisciplinary Center (IDC) Herzliya Review Board approved the study.

### Advertisements

The ads contained text and took the form of a title, a body, and a link to a Uniform Resource Locator (URL) (shown to the users). As noted above, there were 10 different textual versions of the title and body, as shown in Table 1. The ads referred people to one of 3 sites, the names of which were visible to the user. The 3 sites were as follows: the National Eating Disorders Association (NEDA) [27], the National Institutes of Mental Health (NIMH) [28], and the MyProAna website [29].

A full factorial design was used such that all combinations of titlebody and URL were tested (a total of 30 combinations). The ads were shown only to the people who searched from computers located in the United States and who performed searches on the Bing search engine. According to recent estimates, we noted that 21.4% of the US Internet users use Bing [30]. As per the 2010 US Census, the correlation between the number of Bing users per county in the United States and the number of people in that county is *R*^2^=.83 (*P*<.001). Thus, it is estimated that Bing users are a representative sample of the US population, at least in terms of their geographic distribution.

The ads were shown when the searches of users contained one or more of the following 6 keywords: Thinspiration, Thinspo, Proana/Pro Ana, Anorexia, or eating disorder (see [16] and references therein for the use of these keywords). Several advertisers can bid to show ads for the same keywords, and the Bing advertising system chooses which ads to show based on the monetary bids that the advertisers provide. In this experiment, as recommended by the Binge Ads system, the bids were set between zero and US $0.99 per keyword. If the Ads system chose to show one of the experimental ads, one of the 10 ads was randomly chosen with equal probability. The cost cited above is the cost that the investigators paid for each ad clicked by a user.

If one of the study ads were shown, they were displayed either directly underneath the search text (top of the page) or to the right of the search results (right of the page). Sometimes, the Ads system presented our ads in addition to other ads paid for by other advertisers. In those cases, a placement at location 1 meant that the ad was shown as the first ad in the list, and lower locations implied a lower order within the set of ads shown.

As noted above, the Bing Ads system randomly chose which of the 10 ads to show. Each of the 10 ads had the same probability of being displayed. Although it is likely that most users see the ads, only some users click on the ad. The ads were shown between April 19, 2016, and May 11, 2016.

### Advertisement Categorization

We scored the ads according to their expression of the following six attributes: negativity, ambivalence, acceptability, sociality, treatment, and tips. A description of the attributes is given in Table 2. Categorization was used to generalize the ads in the statistical models below. The ads were categorized by 20 nonspecialist people from the crowdsourcing site *CrowdFlowe* r, who were asked to rate each ad on each attribute, according to the description provided for each category (Table 2), on a scale between 1 (lowest) and 7 (highest). The final score for each ad on each attribute was the average of the 20 scores. As a comparison between laypeople and professionals, 13 mental health multidisciplinary workers from a specialist EDs department also rated the ads on these attributes, so as to provide a view on how specialists view the ads.

**Table 1:** The titles and descriptions of the advertisements used in the study.

Title	Body
Diagnosed with ED^a^?	If you need a place to talk about it, you are welcome to our community
Eating Disorders info	Complete information about Anorexia, Bulimia and other Eating Disorders
Looking for help?	Learn how to deal with Eating Disorders
Sick of your weight?	Join us! Connect with those who completely understand!
Suffer from Anorexia?	Can't live with Anorexia anymore, but can’t live without? Click here!
Suffer from ED?	Click here to learn all about eating disorders
Suffer from ED?	Join our community and get all the support you need
Tired of your Anorexia?	Click here to get in touch with those who feel the same
Tired of your Anorexia?	If you want to stop being controlled by your anorexia, click here!
Want to be thinner?	Enter here if you are tired of not being understood

^a^ED: eating disorder.

**Table 2:** Advertisement attributes and their description.

Attributes	The description given to the crowdsourcing workers and to the specialists
Negativity	The ad^a^ expresses a negative view of EDs^b^ or of weight loss
Ambivalence	The ad expresses ambivalence toward EDs or toward losing weight
Acceptability	The ad implies that EDs and weight loss are acceptable practices
Sociality	The ad offers people to become part of a group
Treatment	The ad will appeal to people who want to get treatment
Tips	The ad would appeal to readers who seek weight loss tips

^a^Ad: advertisement.

^b^EDs: eating disorders.

**Table 3:** Keywords used to identify behaviors of interest.

Categories of users’ interest	Keywords
Pro-anorexia/eating disorders	proana^a^ (and its variations), anorexia, anorectic, EDNOS^b^, bulimia, thinspiration, thinspo, ana buddy
MyProAna	MyProAna
Self-harm	cut my, hurt myself, self-harm, self-injury, self-poisoning, hair pulling
Substance abuse	smoke (and its variations), LSD^c^, hash, vape, heroine, inject
Treatment	psychologist (and its variations), clinic, hospital
Depression	hopeless, helpless, depressed (and its variations)
Suicide	suicide, kill myself

^a^Pro-ana: pro-anorexia.

^b^EDNOS: eating disorder not otherwise specified.

^c^LSD: lysergic acid diethylamide.

### Search Behaviors

On the basis of previous work [23,24], we tracked users’ interest before and after the ad displays. Specifically, we extracted all search queries in the following 7 categories: pro-anorexia, MyProAna, self-harm, substance abuse, treatment, depression, and suicide (see Table 3). These behaviors were tracked from one month before the first ad was shown until a month after the last ad was shown for users who were logged into the system and could thus be tracked.

### Analysis

We modeled the likelihood of a user to click an ad using a logistic regression model with the following independent attributes: time of display (date, hour), URL, ad position (top or right and order of display), device on which the ad was shown, and ad categories (as detailed above). We further tested the effect of individual ads on the change in behavior by modeling attributes of the ads and of the users who saw them using a linear regression model. The attributes used to model each ad display were as follows: user attributes (age, gender), whether the ad was clicked, ad location (position and rank), URL, ad text, number of previous ads that the user was shown, ad position (top or right, and order of display), and ad categories (as detailed above, using the scores of the crowdsourced workers).

## Results

### Agreement Between Experts and Laypeople on Advertisement Attributes

We calculated the Spearman correlation between the average score given by the specialist and the average score for each advertisement, as calculated from the scores given by crowdsourcing workers. Although the scores of both groups were highly correlated for acceptability [rho (ρ)=.66, *P*=.038], sociality (ρ=.92, *P*<.05), and reference to treatment (ρ=.82, *P*<.05), they were not highly correlated for how ads are interpreted on negativity (ρ=.61, *P*>.05), ambivalence (ρ=.25, *P*=.48), and tips (ρ=.30, *P*=.40).

#### Campaign Statistics

**Figure 1:**
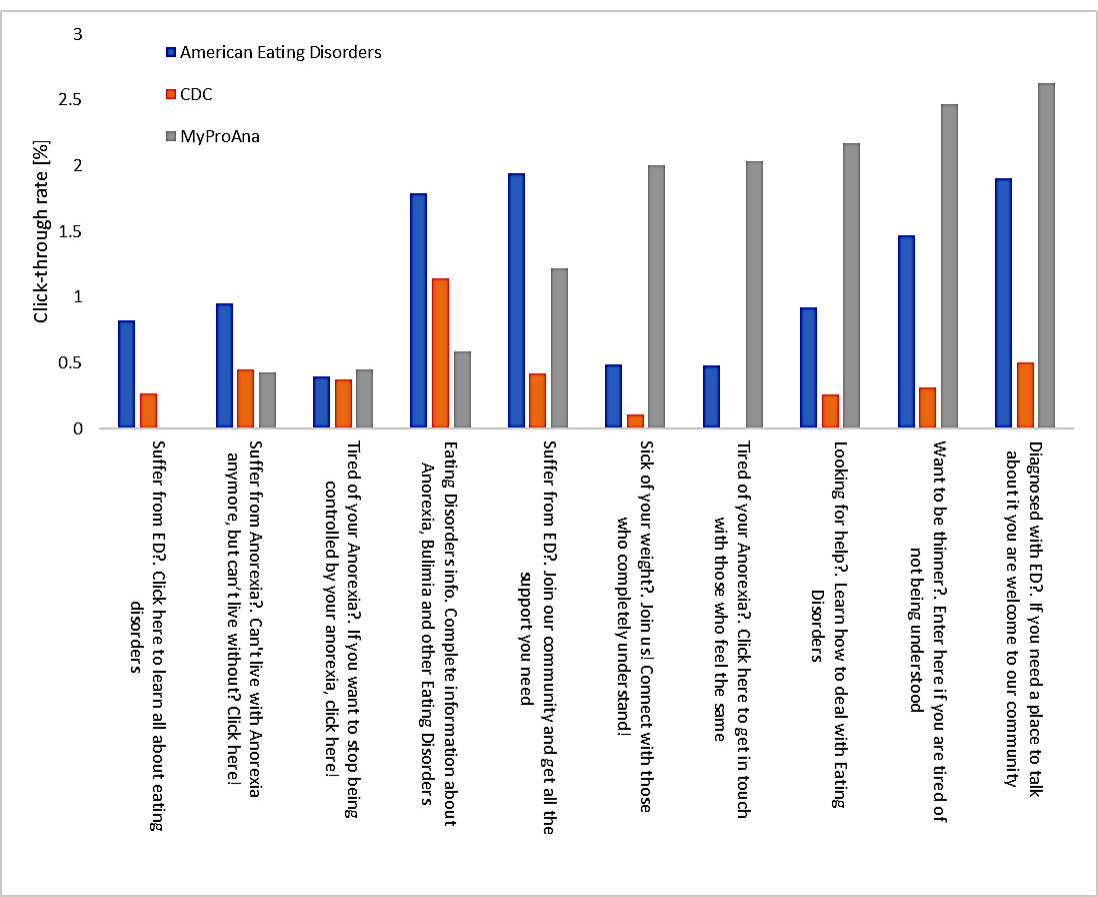
Click-through rates as a function of advertisement text and the site it referred users to. CDC: Centers for Disease Control and Prevention; ED: eating disorder.

The ads were shown 25,554 times and clicked 217 times. Thus, the click-through rate (CTR) was 0.85%, in line with similar advertising campaigns [15]. The CTRs by ad text and the site referred are shown in Figure 1. As Figure 1 shows, CTRs varied considerably within ads—many ads were clicked only when they referred people to one of the 3 sites, but not others.

Note that some people viewed multiple ads (if they made multiple queries), and thus, some of the analysis below is at the level of individual ads, whereas most are at the level of users, taking into account the number of times past advertising was displayed.

**Figure 2:**
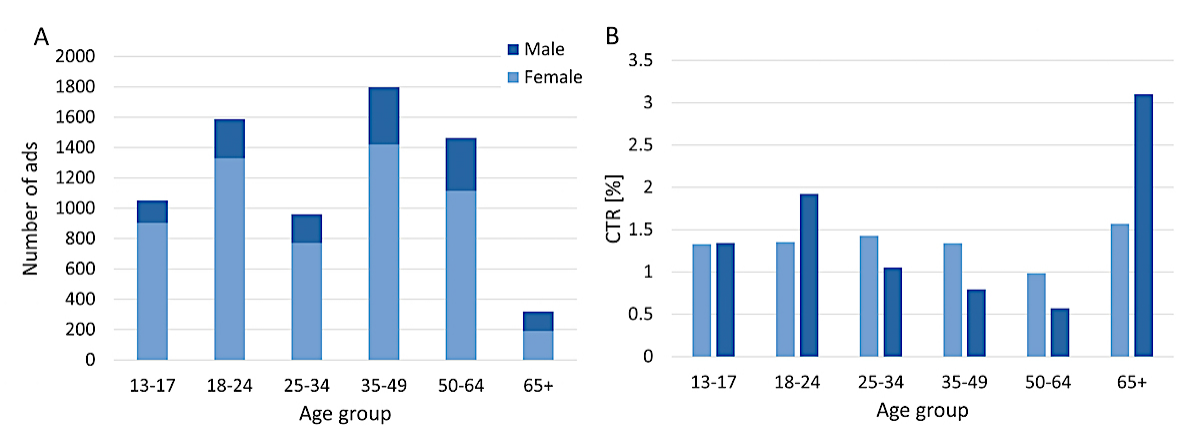
(A) Number of times that advertisements were shown and (B) the click-through rates by age and gender.

The Bing Ads system provides demographic information (age group and gender) for people who saw the ads and provided this information to the Ads system. Figure 2 shows the number of times that the ads were shown, which was contingent on the number of searches, and the CTR by age and gender. Note that some of the users saw multiple instances of the ads. Women were found to be the primary seekers of content using the proposed terms (80% of the ads were shown to women [n=20,443]), and the two age groups with most searches were 18-24 and 35-64 years. CTRs did not vary significantly by age group or gender and were all around 1% (Figure 2).

We modeled the likelihood of a user to click an ad using a logistic regression model with the following independent attributes: time of display (date, hour), URL, ad position (top or right and order of display), device on which the ad was shown, and ad categories (as detailed in the Methods section).

Statistically significant (N=25,554) variables of this model were that the ad referred to NIMH (slope: −1.01, *P*<.001), ad positioned below other ads (slope: −0.35, *P*<.001), ads expressing a negative view of EDs (slope: −0.54, *P*<.001), and ads with a Windows operating system (implying, usually, desktop computers) (slope: 1.2, *P*<.001). Although the first 3 attributes made the ads less likely to be clicked, the last made them more likely to be clicked.

#### Change in Behaviors of Interest

On average, 407 people searched for each of the 7 behaviors of interest. These people made, on average, 2716 queries for each of these behaviors (excluding the queries that triggered the ad display).

We defined an increase in behavior as, *when a user began engaging in a behavior after the ad was shown to them, where this user did not engage in this behavior before the ad was shown, and a decrease as the ceasing search for a behavior after the ad was shown, when the user was searching for it before the ad was shown*. We note that our analysis in this part is based on exposure to the ads and not only for those ads that were clicked. We did it for 2 reasons.

**Figure 3:**
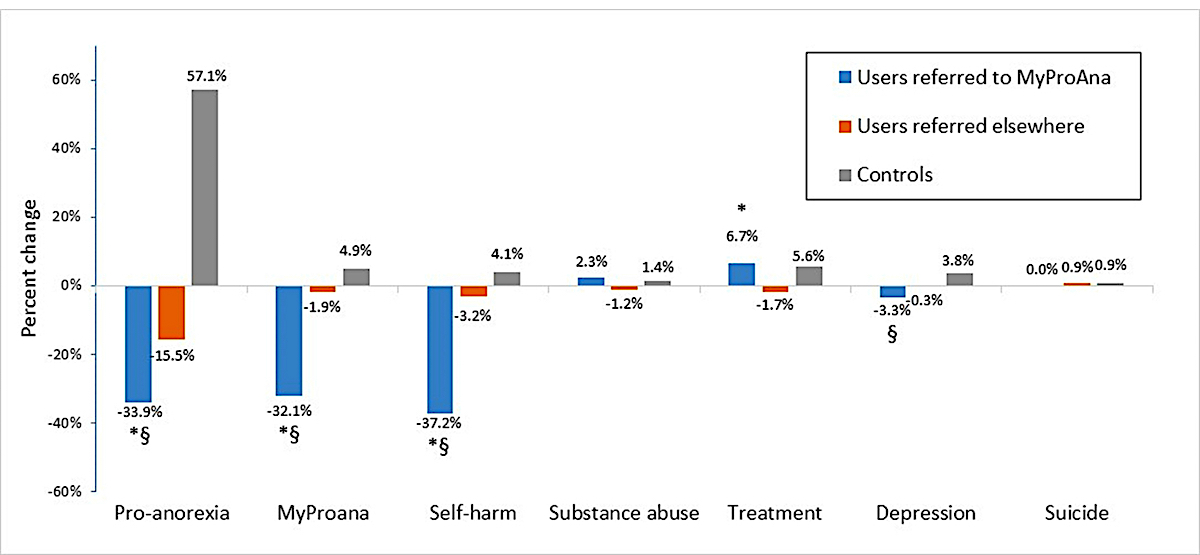
Change in behaviors following ads display. The “*” denotes that the difference between the users referred to MyProAna was statistically significantly different from users referred elsewhere at *P*<.05 (rank sum test). The “§” denotes that the difference between the users referred to MyProAna was statistically significantly different from the controls at *P*<.05 (rank sum test).

First, the number of people who were exposed to ads far exceeds the number of people who clicked on them. Second, previous work [23] has found that exposure is often sufficient to elicit behavioral change. Figure 3 shows the average change in behavior for 3 populations, which were as follows: (1) people who were shown ads referring them to the MyProAna site (n=87); (2) people who were shown ads referring them to one of the two other sites (n=1676), and (3) a random sample of people who made searches containing the same target keywords, but were not shown the ads (n=10,000). Note that no statistically significant difference was found when comparing the 3 populations by age and gender (chi-squared test, *P*>.05).

As Figure 3 shows, people who were referred to MyProAna reduced their search for this website (as can be expected, if they access the site later directly, and not via Bing), but also for pro-ana content and for self-harm content. These reductions were greater than the reductions for users who were referred elsewhere, who experienced the reduction to a smaller extent, and to the control users, who increased their behaviors. Users referred to MyProAna increased their search for treatment, as did control users, who did so to a lesser extent. Users referred elsewhere decreased their searches for this content. Only minor changes were found for the remaining 3 behaviors. Thus, our ads, when they referred people to the MyProAna website, reduced seeking of self-harm and pro-ana content and increased searches for treatment, compared with other populations. We cannot determine whether the individuals directed to MyProAna decreased their search behaviors for pro-ana content and self-harm content because they found their search goal at the MyProAna site or because they did not consume such content anymore. However, given that searches for treatment increased, it is more likely that the latter is true.

We further tested the effect of individual ads on the change in behavior by modeling the attributes of the ads and of the users who saw them using a linear regression model.

**Table 4:** Statistically significant attributes obtained for each behavior. Attributes in quotation marks refer to a specific ad by its title.

Attribute		Slope (SE^a^)	*t* statistic	*P* value^b^
**Pro-ana**^c^**(*R*^2^=.09)**				
	Age	−0.004 (0.001)	−5.66	<.001
	Ad position (rank)	−0.021 (0.010)	−2.03	.04
	“Suffer from ED?”^d^	0.119 (0.039)	3.04	.002
	“Want to be thinner?”	0.233 (0.090)	2.58	.01
**Self-harm (*R*^2^=.13)**				
	Age	0.002 (0.001)	2.40	.02
	Number of previous ads	−0.005 (0.001)	−6.57	<.001
**Substance abuse (*R*^2^=.05)**				
	Referred to MyProAna	0.381 (0.157)	2.42	.02
	“Want to be thinner?”	0.484 (0.209)	2.31	.02
	Number of previous ads	−0.043 (0.012)	−3.44	.001
	Negative view of ED	0.289 (0.098)	2.95	.003
	Treatment	−0.189 (0.090)	−2.10	.04
**Treatment (*R*^2^=.01)**				
	“Want to be thinner?”	0.215 (0.105)	2.05	.04
	Number of previous ads	0.032 (0.001)	3.98	<.001
**Suicide (*R*^2^=.03)**				
	Number of previous ads	0.126 (0.029)	4.28	<.001

^a^SE: standard error.

^b^Statistically significant (*P*<.05) attributes for predicting the change in behavior.

^c^Pro-ana: pro-anorexia.

^d^ED: eating disorder.

The attributes used to model each ad display were as follows: user attributes (age, gender), whether the ad was clicked, ad location (position and rank), URL, ad text, number of previous ads that the user was shown, ad position (top or right, and order of display), and ad categories (as detailed in the Methods section, using the scores of the crowdsourced workers). Statistically significant attributes (*P*<.05) as well as the total explanatory power of the model for each behavior (through the coefficient of determination, *R*^2^) are shown in Table 4. Several observations can be made from Table 4. First, models for pro-ana searches, self-harm, and, to a lesser extent, substance abuse are most predictive, meaning that for those behaviors our models are most accurate in predicting behavioral change. Second, the number of previous ads plays an important role in several models, but not always in the same way; more exposure to our ads results in less self-harm and substance abuse, but more suicide search and treatment queries. Finally, for the pro-ana model, people of lower age were more likely to change their behavior following our ads. The most effective ads were those that either suggested a wish to be thinner or proposed that the person is suffering from their ED.

## Discussion

This study’s results indicate that ads may change the online search behavior of users looking for pro-ana content. Importantly, our study did not find that the ads caused any significant indications of additional harmful online behavior.

Indeed, high exposure to the proposed ads was associated with a decrease in self-harm and substance abuse and an increase in treatment searches. A small increase in suicide-related queries was observed when searchers were repeatedly exposed to ads, where the model explained a very small part of the variance, requiring additional research to validate this possible harm. These finding are in line with previous studies in other areas (eg, smoking cessation), indicating a possible behavioral change based on usage of online ads [31-33]. Interestingly, when an ad referred people to the MyProAna website, it had a beneficial influence, as observed by fewer harmful searches. Specifically, among those referred to MyProAna, there was a reduction in the searches for pro-anorectic content as well as self-harm content. This is especially impressive as the control group (which was not presented with the experiment ads) increased their behavior. This study suggests that self-harm online behaviors could be changed using simple and cost-effective online ads.

One explanation for the decrease in pro-ana and self-harm queries might be that people referred to the pro-ana website found information on these topics within the website. However, this explanation is neither congruent with the reduction in pro-ana searches in people who were referred to NIMH and the Eating Disorders Association website nor the increase in treatment searches. Thus, we can hypothesize that the change in searches may be reflective of the user’s satisfaction related to information quality, feelings of being understood, and feeling of support and community adherence.

The most effective ads were those that addressed both the addictive search for thinness and control and the emotional suffering and distress. Moreover, varied ads are required for a successful campaign, which can elicit behavioral change. In addition, ads referring to NIMH and those with negative views of ED were less likely to be clicked. We hypothesize that the use of a complex motivational approach addressing both the irresistible addiction and the emotional suffering through the ad, instead of the standard psychoeducational health-oriented nondialectic wording, changes the search behavior. This is in line with the finding [33,34] which indicated that emotional ads are more effective than educational ads. Moreover, when targeting users showing an interest in EDs, it is important to use various ads that can elicit behavioral change. These varied ads may influence different types of populations dealing with EDs or populations that hold both aspects of addiction and distress. Interestingly, users and ED specialists only partially agreed on some aspects of the ads. This finding is in line with the previous studies showing that people dealing with ED issues or patients suffering from ED view ED topics differently from professional specialists [35]. It can be hypothesized that specialist ED workers interpret the ads adopting the role of a professional motivated to encourage change and are less *objective* than laypersons when characterizing the different meanings of the ads.

The main limitation of the study is that changing online searches does not necessarily mean real behavior changes offline. Moreover, this study did not include an actual model of behavior change. Though past studies have found that online behavior corresponds to offline behavior, this is not assured to happen in all cases. In addition, the study examined the ads and not the sites. This study was unable to statistically differentiate between users who were presented with the ads and those who also entered the websites via the provided links. Future studies should aim to differentiate between the two groups. Another limitation is that the keywords chosen to trigger the ads can be used by both people suffering from a ED and ones with a passing interest in them, and it is possible that more targeted forms of advertising can be even more effective. Therefore, any search changes we observed are likely to underestimate the true power of the ads. Finally, our demographic data were based on self-reports by part of the sample included.

Given these limitations, this study has important clinical implications. The main one is that a cost-effective use of online ads may change a user’s searches and website visits. Users searching for pro-ana content or visiting pro-ana websites can potentially be redirected to search healthier content and to visit less harmful websites.

Our findings suggest that people preoccupied with ED issues, those who are suffering from an ED, and those who have the potential to develop an ED can be redirected to less pathological online searches when appropriate pathways are offered. However, sites should not be focused solely on providing formal information and preaching an urgent change. Future work will focus on new ways to attract users searching for ED content. These new ways require the acceptance of ambivalence and encouragement of self-disclosure, dialogue, and empathic support.

## Appendix

Multimedia Appendix 1 CONSORT‐EHEALTH checklist (V 1.6.1).

## Abbreviations

BT: behavioral targeting
CDC: Centers for Disease Control and Prevention
CTR: click-through rate
EDNOS: eating disorder not otherwise specified
EDs: eating disorders
IDC: Interdisciplinary Center
LSD: lysergic acid diethylamide
NEDA: National Eating Disorders Association
NIMH: National Institutes of Mental Health
Pro-ana: Pro-anorexia
RCT: randomized controlled trial
SE: standard error
URL: Uniform Resource Locator
